# Clinical results with two different pharmaceutical preparations of riboflavin in corneal cross-linking: an 18-month follow up

**DOI:** 10.1186/s40199-015-0091-z

**Published:** 2015-01-24

**Authors:** Hassan Hashemi, Mohammad Amin Seyedian, Mohammad Miraftab, Hooman Bahrmandy, Araz Sabzevari, Soheila Asgari

**Affiliations:** Noor Ophthalmology Research Center, Noor Eye Hospital, No. 96 Esfandiar Blvd., Vali’asr Ave, Tehran, Iran; Department of Epidemiology and Biostatistics, School of Public Health, Tehran University of Medical Sciences, International Campus (TUMS-IC), Tehran, Iran

**Keywords:** Keratoconus, Cross linking, Riboflavin, Sina Darou, Streuli Pharma, Clinical trial

## Abstract

**Background:**

Comparison of long-term clinical results of two different pharmaceutical formulations used in corneal cross-linking (CXL) in keratoconus patients.

**Methods:**

Sixty eyes of 60 keratoconus patients underwent CXL in two groups. We used riboflavin preparations from Sina Darou, Iran in group A, and Streuli Pharma, Switzerland in group B. Here we made inter-group comparison of changes in vision, refraction, Pentacam indices, corneal biomechanical indices, and endothelial cell count (ECC) 18 months after CXL.

**Results:**

Since four patients were lost to follow-up, 56 eyes (28 eyes in each group) were compared. Mean improvement in uncorrected visual acuity (UCVA) was 0.31 ± 0.65 LogMAR (P = 0.014) in group A and 0.24 ± 0.62 LogMAR (P = 0.082) in group B. Best corrected visual acuity (BCVA) remained quite unchanged in both groups (P = 0.774). Mean spherical refractive error reduced by 0.45 ± 1.15 diopter (D) (P = 0.041) in group A and 0.27 ± 1.73 D (P = 0.458) in group B (P = 0.655). Cylinder error and spherical equivalent had a similar trend without any change. Max-K (P = 0.006) and mean-K (P = 0.044) decreased significantly more in group A compared to group B. The reduction in CCT was significantly more in group A than group B (P = 0.004). Q-value was quite unchanged in both groups (P = 0.704). The inter-group difference in CH reduction was borderline significant statistically (P = 0.057). Changes in corneal resistance factor and endothelial cell count were not significantly different between two groups (P = 0.117 and P = 0.229).

**Conclusion:**

Clinical results of CXL with the domestic preparation of riboflavin are similar to that achieved with the Swiss made product in some aspects, and it is the preferred brand in some other aspects. This study will continue to report longer follow-up results.

**Trial registration:**

IRCT201212034333N2

## Background

Collagen cross linking with riboflavin (CXL) was first developed by Wollensak et al. [1] to stop the progression of keratoconus. In this procedure, riboflavin plays an important role because it absorbs UVA and it reduces cell damage [1]. The riboflavin preparation used in Iran is a product of Streuli Pharma, a Swiss company. Export company of Sina Darou has manufactured this product in Iran with the same formulation and amount of active substance as the Swiss equivalent, and we have studied its clinical results in patients treated with CXL. In the preliminary report [2], we demonstrated that 6-month changes in vision, refraction, K-reading, corneal biomechanics, and endothelial cell count parameters were not significantly different between the two groups, and clinical results achieved with these formulations are similar. Here we compare 18-month results between these two preparations, so that we can comment on their clinical use with better certainty.

## Methods

The complete study methodology has previously been described [2]. In brief, we enrolled 60 eyes of 60 keratoconus patients (30 eyes in each group) in this parallel non-randomized clinical trial. The Iranian preparation of riboflavin 0.1% (Sina Darou, Iran) was used in the first group (group A), and the Swiss preparation of riboflavin 0.1% (Streuli Pharma, Uznach, Switzerland) was used in the second group (group B) during the procedure. Inclusion criteria were the diagnosis of progressive keratoconus on clinical exam which is confirmed paraclinically, age between 15 and 35 years, keratometry less than 55.0 diopter (D), and a minimum central corneal thickness (CCT) of 400 microns (μm).

First the study methods and objectives were explained to the subjects, and they were enrolled in the study after obtaining written informed consents. The study was approved by Noor Review Board. Iranian Registry of Clinical Trials also approved the study (registration number: IRCT201212034333N2).

The surgical procedure has already been described [3]. After local anesthesia, 3 or 4 strips 2 millimeter wide, and about 1 millimeter apart were removed from the central 7 millimeter of the cornea, leaving the corneal epithelial intact in-between. Another epithelium strip was removed horizontally from the inferior third of the cornea. Then, riboflavin 0.1% drops in 20% dextran were instilled onto the corneal surface for half an hour at 3 minute intervals. After ensuring of the presence of riboflavin and observing a yellow Tyndall effect in the anterior chamber, irradiation at a wavelength of 370 nanometer and power of 3 mW/cm^2^ was commenced from a distance of 5 cm. Irradiation was done using the UVX system (IROC, Zürich, Switzerland). Riboflavin instillation continued every three minutes during the 30 minutes of irradiation. At the end of this stage, the corneal surface was rinsed with sterile balanced saline solution, a soft bandage contact lens (Night & Day, Ciba Vision, Duluth, GA) was applied, and chloramphenicol 0.5% eye drop was instilled. Postoperative medication included chloramphenicol 0.5% eye drops four times daily, betamethasone 0.1%, and preservative free artificial tears (Hypromelose) as required. Patients were examined on day 1 and 3 after the procedure, and the lens was removed the epithelium had healed. After removing the lens, chloramphenicol was discontinued, and betamethasone was continued twice daily for another week. When the epithelium was not healed, daily visits were continued until complete healing. No case of intraoperative or postoperative complication was observed.

Paraclinical tests included the assessment of uncorrected and best spectacle corrected visual acuity (UCVA and BCVA) using the Snellen chart, and determining the spherical equivalent (SE) using a Retinoscope (HEINE BETA 200, Germany). We also checked corneal topographic indices using Pentacam (Oculus Optikgerate GmbH, Germany), corneal biomechanical parameters using the Ocular Response Analyzer (ORA; Reichert Ophthalmic Instruments, Buffalo, USA), and the endothelial cell count (ECC) with a non-contact specular microscope (Konan Medical, Hyogo, Japan).

The trend of changes was compared between the two groups using repeated measures analysis of variance, and intra-group differences between before and 18 months after the procedure was assessed using the paired t test. We chose a significance level of 0.05.

## Results

Since 2 patients from each group did not show up on the 18 month follow-up exam, 56 eyes of 56 keratoconus patients treated with CXL (28 eyes in each group) were compared. Their mean age was 24.32 ± 4.59 years, and 65% were male. Patients were treated with Iranian riboflavin (group A) and Swiss riboflavin (group B) in two groups of 28 people. Since the study had a non-randomized approach, preoperative values of all parameters were compared between the two groups, and there was no significant difference in any case.

At 18 months, mean UCVA improved similarly (P = 0.684) by 0.31 ± 0.65 LogMAR (P = 0.014) in group A and 0.24 ± 0.62 LogMAR (P = 0.082) in group B. BCVA remained unchanged in both groups (P = 0.774). Mean spherical refractive error reduced by 0.45 ± 1.15 D (P = 0.041) in group A and 0.27 ± 1.73 D (P = 0.458) in group B (P = 0.655). Cylinder error and spherical equivalent had a similar trend without any change (Table 1).

**Table 1:** **Trend of changes in vision and refraction parameters in the two groups of keratoconus patients treated with Iranian and Swiss preparations of riboflavin**

	**Riboflavin**	**No of eyes**	**Pre operation**	**6 months after surgery**	**18 months after surgery**	**P-value** ^*^	**P-value** ^**^
UCVA (logMAR)	Sina Darou, Iran	28	0.77 ± 0.66	0.45 ± 0.36	0.44 ± 0.41	0.014	0.684
	Streuli Pharma, Switzerland	28	0.89 ± 0.56	0.79 ± 0.53	0.66 ± .47	0.082	
BCVA (logMAR)	Sina Darou, Iran	28	0.20 ± 0.19	0.19 ± 0.13	0.17 ± 0.13	0.710	0.774
	Streuli Pharma, Switzerland	28	0.22 ± 0.20	0.20 ± 0.23	0.22 ± 0.22	0.880	
Sphere (diopter)	Sina Darou, Iran	28	−1.36 ± 2.18	−1.42 ± 2.36	−1.15 ± 2.25	0.041	0.655
	Streuli Pharma, Switzerland	28	−1.69 ± 1.92	−1.73 ± 2.48	−1.59 ± 2.69	0.458	
Cylinder (diopter)	Sina Darou, Iran	28	−2.67 ± 1.83	−2.36 ± 1.79	−2.40 ± 1.77	0.827	0.642
	Streuli Pharma, Switzerland	28	−2.64 ± 1.91	−2.95 ± 1.97	−2.33 ± 2.16	0.332	
Spherical equivalent (diopter)	Sina Darou, Iran	28	−2.69 ± 2.44	−2.60 ± 2.85	−2.35 ± 2.52	0.093	0.875
	Streuli Pharma, Switzerland	28	−3.01 ± 2.29	−3.20 ± 2.80	−2.76 ± 3.12	0.230	

*Intra-group comparison of parameters before and 18 months after the procedure using paired t test.

**Inter-group comparison of parameters’ trend of changes using repeated measures ANOVA.

Despite similar 6 month trends between the two groups, the 18-month decrease in max-K was 1.44 ± 1.31 D (P < 0.001) in group A and 0.52 ± 0.82 D (P = 0.007) in group B, and the inter-group difference in was statistically significant in this regard (P = 0.006). Mean-K decrease was 1.33 ± 1.19 (P < 0.001) and 0.69 ± 1.00 (P = 0.004) in groups A and B, respectively; the difference was statistically significant (P = 0.044). CCT decreased significantly more (P = 0.004) in group A (47.00 ± 33.10 μm, P < 0.001) than group B (22.32 ± 20.87 μm, P < 0.001). Q-value remained quite unchanged in group A and became slightly prolate in group B; the inter-group difference was not statistically significant (P = 0.704) (Table 2).

**Table 2:** **Trend of changes in parameters measured with Pentacam compared between two groups of keratoconic patients treated with Iranian vs. Swiss preparations of riboflavin**

	**Riboflavin**	**No of eyes**	**Pre operation**	**6 months after surgery**	**18 months after surgery**	**P-value** ^*^	**P-value** ^**^
Maximum keratometry (Diopter)	Sina Darou, Iran	28	49.05 ± 3.57	48.45 ± 2.79	47.74 ± 3.76	<0.001	0.006
	Streuli Pharma, Switzerland	28	48.60 ± 3.33	48.74 ± 3.56	48.06 ± 3.16	0.007	
Mean keratometry (Dipoter)	Sina Darou, Iran	28	47.14 ± 3.37	46.37 ± 2.30	45.98 ± 3.78	<0.001	0.044
	Streuli Pharma, Switzerland	28	47.07 ± 2.91	47.00 ± 3.21	46.37 ± 3.00	0.004	
Q-value	Sina Darou, Iran	28	−0.69 ± 0.38	−0.68 ± 0.39	−0.68 ± 0.57	0.651	0.704
	Streuli Pharma, Switzerland	28	−0.72 ± 0.33	−0.75 ± 0.45	−0.63 ± 0.38	0.064	
Central corneal thickness (μm)	Sina Darou, Iran	28	482.1 ± 29.7	467.5 ± 29.8	441.5 ± 45.0	<0.001	0.004
	Streuli Pharma, Switzerland	28	496.9 ± 35.6	481.5 ± 37.3	474.7 ± 41.2	<0.001	

*Intra-group comparison of parameters before and 18 months after the procedure using paired t test.

**Inter-group comparison of parameters’ trend of changes using repeated measures ANOVA.

The inter-group difference in corneal hysteresis (CH) decrease was borderline significant (P = 0.062). Corneal resistance factor (CRF) decrease was not significantly different between the two groups (P = 0.242). Mean ECC decreased similarly in both groups (P = 0.598) (Table 3).

**Table 3:** **Trend of changes in corneal biomechanical parameters and endothelial cell count compared between two groups of keratoconic patients treated with Iranian vs. Swiss preparations of riboflavin**

	**Riboflavin**	**No of eyes**	**Pre operation**	**6 months after surgery**	**18 months after surgery**	**P-value** ^*^	**P-value** ^**^
Corneal hysteresis (mmHg)	Sina Darou, Iran	28	7.67 ± 1.44	6.69 ± 1.52	6.44 ± 1.37	<0.001	0.062
	Streuli Pharma, Switzerland	28	7.63 ± 2.12	7.36 ± 1.50	7.53 ± 1.66	0.701	
Corneal resistance factor (mmHg)	Sina Darou, Iran	28	6.74 ± 1.66	6.20 ± 1.24	5.88 ± 1.93	0.023	0.242
	Streuli Pharma, Switzerland	28	6.94 ± 1.97	6.94 ± 1.74	6.79 ± 1.90	0.716	
Endothelial cell count (cell/mm^2^)	Sina Darou, Iran	28	2789.3 ± 160.8	2455.4 ± 312.9	2511.7 ± 271.8	<0.001	0.176
	Streuli Pharma, Switzerland	28	2731.6 ± 262.9	2470.7 ± 274.1	2574.9 ± 305.5	<0.001	

*Intra-group comparison of parameters before and 18 months after the procedure using paired t test.

**Inter-group comparison of parameters’ trend of changes using repeated measures ANOVA.

## Discussion

CXL slows down or halts the progression of keratoconus by forming covalent bonds in the corneal stroma that are created as an effect of free radicals. In this process, UV-irradiated riboflavin produces free radicals, and riboflavin concentration influences the level of UV absorption and strengthening reactions in the cornea. In-vivo, riboflavin can increase UV absorption up to 95% [4]. This is while UV absorption in the cornea is only 25-35% without riboflavin [5]. With riboflavin concentrations between 0 to 0.04%, UV absorption increases linearly, but has no further effect [6]. Thus, using riboflavin is one of the main pillars of the treatment.

In the 6-month report [2], we compared preliminary clinical results of treatment with Iranian and Swiss preparations of riboflavin which demonstrated the effectiveness of the Iranian preparation. Six-month changes in vision, refraction, corneal topographic and biomechanical parameters, and ECC were similar in the two groups, and no inter-group difference was found. Clinical parameters were not significantly different at 18 months either, and both preparations were similar in terms of stopping the progression of keratoconus. The two preparations were only different in terms of corneal topographic indices; better flattening was achieved with the Iranian preparation while CCT decrease was less with the Swiss product. It must be noted, however, that this study was nonrandomized, and to lessen the effect of this limitation, we performed matching using base indices.

Stability of vision and refraction parameters in the group treated with Sina Darou riboflavin indicated that disease progression had stopped. Various results have been reported after treatment with CXL. Some studies demonstrated no change [2,7,8], some observed improvement [8-10], and some showed reduced vision and increased refraction [7]. This can be due to inter-study differences in preoperative values or disease severity in the study samples. Different corneal structures in different populations can be another reason that causes such differences. Another point is that vision assessment is a subjective test which can be influenced by environmental conditions, optometrists’ accuracy, and patients’ condition. Thus, diverse results can be expected.

Corneal topographic changes were significantly different between the two groups. Although both groups demonstrated a significantly reduced protrusion and decreased CCT, patients achieved better corneal flattening when treated with Iranian riboflavin. The reduction in corneal thickness, however, was less in the group of patients treated with Swiss riboflavin. This could imply better intra-fibril bond formation is supported by the Iranian preparation due to better UVA absorption, and thus, keratometry is decreased. The lack of significant inter-group difference in ECC showed that despite better UV absorption, cytotoxic effects were not intensified, and there was no keratocyte loss [11,12]. Some studied have demonstrated reduced corneal thickness despite reduced keratometry and halted disease progression [13-15], and this has mostly been attributed to stages of epithelium removal and riboflavin instillation [16].

CRF reduction was similar in the two groups. The inter-group difference in CH reduction was borderline significant (P = 0.062). However, CH and CRF are not enough to show changes in corneal biomechanical properties [17], we would need to examine other indices measured with ORA to have a more accurate assessment of the effects of these two preparations.

## Conclusion

Finally, based on 18-month results, apart from better flattening with the Iranian preparation and better maintenance of corneal thickness with the Swiss product, cresults in terms of clinical vision, refraction, biomechanical properties, and the endothelial cell count were comparable with these two types preparations of riboflavin. We can thus conclude that the Iranian riboflavin (Sina Darou) can be an alternative for its Swiss counterpart in CXL. This study will continue to assess the stability of results at later follow-ups.

## Abbreviations

CXL: Corneal cross linking
SE: Spherical equivalent
BCVA: Best corrected visual acuity
UCVA: Uncorrected visual acuity
max K: Maximum keratometry
CCT: Central corneal thickness
ECC: Endothelial cell count
CH: Corneal hysteresis
CRF: Corneal resistance factor
